# AGE, EXPERIENCE OF RACIAL MICROAGGRESSIONS, AND DISTRESS

**DOI:** 10.1093/geroni/igz038.2613

**Published:** 2019-11-08

**Authors:** Shayla Thompson, Broderick Sawyer, Suzanne Meeks

**Affiliations:** University of Louisville, Louisville, Kentucky, United States

## Abstract

Racial microaggressions are a common form of racial discrimination consisting of subtle or interpersonal slights. Racial microaggressions are linked to various kinds of psychological distress in younger adults, but have not been studied across the lifespan. We examined the relationship of racial microaggressions with psychological distress and anger rumination among younger and older adults identified as racial or ethnic minorities. We hypothesized that age would moderate the relationship between racial microaggressions and psychological distress and anger rumination, that is, the relationship would be weaker for older than for younger adults. Participants were recruited from Amazon Mechanical Turk and were compensated $1 for their participation. Preliminary tests of the hypotheses (N=220), using multiple regression analyses to test for moderation, failed to support the hypothesis that age would mitigate the impact of microaggressions on symptom severity. Both age and microaggressions were related to psychological distress and anger rumination, but contrary to prediction, older adults showed more exacerbation of distress in the face of microaggressions than younger adults. The results also differed by gender and ethnic groups, suggesting the importance of examining intersectional experiences of race, gender, and age in response to discrimination. These cross-sectional findings lend support to the importance of considering both subtle and overt discriminatory experiences in understanding the mental health challenges for minority groups in the U.S., but more work is needed to examine the intersection of ethnicity with other demographic variables, and to understand how the lifelong experiences of discrimination may shape older adults’ vulnerability, well-being, and resilience.

